# ‘I think it depends how it’s done’: a qualitative study of screening attendees’ perspectives on receiving physical activity advice within UK NHS cancer screening programmes

**DOI:** 10.1136/bmjopen-2025-099416

**Published:** 2025-11-21

**Authors:** James Murphy, Claire Stevens, Anna L Roberts, Charlotte Vrinten, Jo Waller, Samuel G Smith, Rebecca J Beeken

**Affiliations:** 1 University of Leeds, Leeds, UK; 2 University College London, London, UK; 3 Imperial College London, London, UK; 4 Queen Mary University of London, London, UK

**Keywords:** ONCOLOGY, QUALITATIVE RESEARCH, Mass Screening

## Abstract

**Abstract:**

**Objectives:**

Cancer screening appointments are an opportunity to encourage positive behavioural changes. Up to 80% of cancer screening attendees are open to discussing physical activity during cancer screening, but some say this would deter them from future screening. This study aimed to gain an in-depth understanding of individuals’ receptivity to physical activity advice at cancer screening.

**Design:**

Interview-based qualitative study.

**Setting and participants:**

The study was conducted from May 2017 to September 2018 in the UK. Participants were recruited using adverts on two university campuses, Facebook and a participant recruitment agency. To be eligible, participants had to have an upcoming cancer screening appointment within 2 weeks. There were 30 participants.

**Procedures:**

Participants recorded their receptivity to physical activity advice in the days before and after screening. Data-prompted semi-structured interviews explored these responses. Interviews were analysed using a thematic framework analysis.

**Results:**

Participants felt discussing physical activity at cancer screening would be relevant. However, participants experienced anxiety related to the screening process which could increase or decrease their receptivity. Participants felt if information was delivered in a judgemental way, it could negatively impact future screening participation.

**Conclusions:**

Screening attendees’ receptivity could be influenced by the timing of a discussion and by their levels of anxiety throughout screening. Participants’ anxiety during screening can either reduce their ability to engage in a discussion or increase the relevance of the discussion. The communication style of the healthcare practitioner was key for why some screening attendees could be deterred from future cancer screening.

**STRENGTHS AND LIMITATIONS OF THIS STUDY**

- This study used data prompted interviews to generate rich qualitative data on cancer screening attendees’ receptivity to physical activity advice.
- Participants were recent cancer screening attendees, reducing recall bias and enhancing ecological validity.
- Self-selection into the study may have resulted in a sample more receptive to physical activity advice than the general population.
- Participants’ views were considered collectively, and potential differences by gender or screening programme (breast, cervical, bowel) were not explored.

## Introduction

 Meeting the UK National Health Service (NHS) physical activity guidelines of at least 150 minutes of moderate intensity physical activity per week (being physically active) reduces a person’s risk of developing multiple types of cancer.^1^,^5^ However, one-third of adults in the UK are not physically active.^6^ National guidelines recommend healthcare practitioners discuss physical activity in every patient contact to try to increase rates of physical activity.^7^

Cancer screening programmes in England bring over 5 million people into contact with a healthcare professional every year.^8^ This figure was higher before bowel cancer screening transitioned to faecal occult blood testing. Cancer screening appointments could be an ideal time for healthcare professionals to discuss cancer risk-reducing behaviours because they are thought to be a teachable moment.^9 10^ A teachable moment is a time when people are more likely to consider changing their behaviours. This can occur following a health event when the person considers the link between their current behaviour and their health.^11^ The teachable moment heuristic model proposes that a change in perceived risk to health, or an affective response to a health event, prompts changes in behaviour.^11^ While participating in cancer screening, people may be thinking more about their risk of cancer and considering their current health behaviours. Therefore, it is thought that discussing behavioural changes that could reduce a person’s risk of cancer, such as physical activity, may be particularly effective if delivered alongside cancer screening.^9 10^ There is evidence that addressing lifestyle risk factors such as alcohol consumption, smoking, diet and obesity alongside cancer screening is acceptable to patients as well as effective.^12^,^15^ However, there is less published research examining the acceptability of physical activity advice alongside cancer screening. Previous quantitative studies suggest up to 80% of people attending cancer screening are open to receiving behaviour change advice alongside screening.^1216^,^18^ However, a minority report that receiving this advice alongside screening would deter them from attending further cancer screening appointments.^12 16 17 19^ It is important to understand more about variation in the acceptability of behaviour change advice to ensure future behaviour change initiatives do not reduce the effectiveness of NHS screening programmes.

Therefore, the aims of this study were to gain an in-depth understanding of individuals’ receptivity to physical activity advice at cancer screening, and how personal experiences of screening may influence preferences for receiving this advice.

## Methods

### Design and setting

This qualitative interview-based study was embedded within a mixed-methods study (‘Conversation Time’), which aimed to assess openness to a conversation about physical activity around the time of cancer screening.^20^ The study took place in the UK.

### Recruitment and participants

For ‘Conversation Time’, participants were recruited by adverts posted on Facebook and the ‘Call for Participants’ website, and posters were placed around two university campuses. Participants were also recruited by a specialist participant recruitment agency (Saros Research) which emailed the details of the study to their participant database.

The ’Conversation Time’ study used Ecological Momentary Assessment (EMA), which is a method of capturing repeated measurements of behaviour in real-time.^21^ Potential participants were eligible to take part in ‘Conversation Time’ if they had an upcoming NHS breast, bowel or cervical cancer screening appointment within 2 weeks of joining the study. This time limit was set to include participants who had recent experience of cancer screening and to allow sufficient time for the study procedures prior to the cancer screening appointment. Bowel cancer was included because recruitment took place when bowel cancer screening was still a face-to-face appointment (a flexible sigmoidoscopy). Exclusion criteria included: a previous diagnosis of the cancer that they were attending screening for, not understanding English or not having access to a smartphone.

To be eligible for this study, participants had to have attended their cancer screening appointment and be available for a >30 minute interview over the telephone or in person. Recruitment took place between May 2017 and September 2018. The participants were not known to the researchers prior to this.

Participants in ‘Conversation Time’ were invited to complete a survey five times per day, for 11 days (5 days prior to a screening appointment, on the day of screening, and the 5 days post appointment) using a mobile phone application.^20^ A key question within this survey, at each time point, asked them to report their openness, on a rating scale from 0 to 10, to having a conversation with a healthcare professional about physical activity and cancer prevention. This approach captured fluctuations in receptivity across different moments and contexts, rather than a single static measure. As openness varied within individuals over time, it was not summarised into a single score or categorised per participant.

All participants who completed the ‘Conversation Time’ study and who had attended screening were invited to participate in this study. Participants were informed that the purpose of the interview was to explore their experience of cancer screening and that they were contributing to an educational project. Participants were consented to these interviews separately. Participants were given a small monetary gift, of approximately £20, as an acknowledgement of the time taken to participate in this research.

### Procedures

A semi-structured interview topic guide was created. In the second part of this, participants were asked about their openness to having a conversation about physical activity at their cancer screening appointment (online supplemental appendix A), which was supplemented by each participant’s EMA data from the initial survey. Prior to the interview, participants were provided with a summary of their data as a visual memory aid to guide the discussions. Providing participants with EMA data enables data prompted interviews (DPIs) in which these data can guide discussion and help participants understand and articulate processes which may change over time.^22^ DPIs using EMA data can help researchers to understand between-person variation in an outcome in line with our research question.

Interviews were conducted within 3 weeks of the participant’s cancer screening appointment. The majority were over the phone, and two were conducted in person (one at University College London and one at a meeting room close to the person’s home). Only the interviewer (CS, Female, PhD student with a background in behavioural sciences) and the participant were present during the interviews. All interviews were recorded and transcribed verbatim. The consolidated criteria for reporting qualitative research checklist was used to ensure comprehensive reporting of the study (online supplemental appendix B).^23^ In the results, theme frequency is not reported. This is because, by providing quantitative values alongside the qualitative data, undue weight could be given to particular themes when their value should be based on the theme’s significance in answering the research question.^24^

### Data analysis

The analysis of the interview data was informed by the teachable moment heuristic.^11^ However, new ideas were also generated inductively. The framework method of data management was used and a thematic analysis performed.^25 26^ Initially, one researcher (CS) re-read transcripts and notes made during the interviews. After familiarisation, notes were made of recurring codes and themes which were used to develop an analytical framework in Microsoft Excel (online supplemental appendix C). The data were then summarised within the analytical framework. At this stage, a second researcher (ALR, Female, PhD student) reviewed a random sample of transcripts (20%) and provided an additional summary of the data. Summaries were compared between researchers, and the initial analytical framework and associated themes were refined through discussion. These themes were then explored and confirmed by a third researcher (JM, Male, medical doctor). Data saturation was not assessed.

## Results

### Sample characteristics

Of the 41 participants involved in ‘Conversation Time’, 30 agreed to take part in this study. Of the 30 participants, 4 had been recruited via the adverts placed online and on university campuses, and 26 were recruited via the specialist participant recruitment agency. Interviewed participants were recent cervical (n=17, 57%), breast (n=10, 33%) and bowel (n=3, 10%) screening attendees. Most were female (n=28, 93%) and of White ethnicity (n=27, 90%). The mean age of the sample was 44 years old (range 27–69). One in five participants had taken part in the cancer screening programme for the first time. Sample characteristics are presented in table 1.

**Table 1:** Sample characteristics (n=30)

Characteristic	
Age (mean (range))	44.4 (27–69 years)
Sex (female)	93.3 (28)
Educated to degree level or above	66.7 (20)
Ethnicity (white)	90.0 (27)
First time participating in screening programme	20.0 (6)
Screening programme	
Cervical	56.7 (17)
Breast	33.3 (10)
Bowel	10.0 (3)

Data are n (%) unless otherwise indicated.

The results are presented within themes which highlight key reasons for differences in participants’ receptivity to a conversation about physical activity during cancer screening. Direct quotes to support the results are included.

### Relevance and convenience

Participants who said they were receptive to a physical activity discussion during cancer screening felt that the cancer screening appointment was a convenient time to discuss this topic for a few reasons. First, participants reported thinking more about their risk of cancer during the screening appointment and could feel particularly at risk of developing cancer around that time.

I think, just the process of going through this (cancer screening) pricks your ears up a bit… Because I was going to have this done, it was making me think about it (cancer) (Bowel screening, Female, #16)

Participants felt cancer screening was, therefore, a good time to discuss cancer risk-reducing behaviours such as physical activity because they were thinking about reducing their risk at that time. After completing screening, some participants reported thinking less about their risk of cancer, and so felt the relevance of discussing physical activity was reduced.

I think the best time would be during your appointment… because you’re there anyway… you are probably thinking about it a bit more… I think after the appointment, there’s… a feeling of right, that’s done with now, I can… forget it*.*(Cervical screening, Female, #6)

Second, participants thought it was convenient to discuss physical activity during a cancer screening appointment because they were already present in a healthcare setting with a healthcare practitioner. Participants also felt they would prefer to discuss physical activity during the cancer screening appointment rather than during other visits to a healthcare practitioner when they may be feeling unwell.

I think, after you’ve had the screening … I think that’s a good time, ‘cause you’re there already. You’re not there because you’re ill, or anything… so you’re probably a bit more open to… having a conversation. (Cervical screening, Female #12).

### Anxiety during screening

Attending a cancer screening appointment could cause participants anxiety about the examination part of the cancer screening or about their risk of cancer. First-time attendees were particularly anxious about the examination. Because of this anxiety, some participants felt strongly that physical activity should not be discussed during the screening appointment.

I would probably be not a very happy person… You’re nervous enough as it is and then to have someone tell you what you should have been doing, when you weren’t doing… it’s just crazy. (Cervical screening, Female, #14)

These participants explained they felt so anxious during the screening appointment that they would not be able to process any advice given at that time.

I was too focussed—on being convinced I was, I was going to get, or I had, cancer. Um, and that I wouldn’t have been, um, open to listening to anybody about that, on that particular day. (Breast screening, Female, #17)

Conversely, some participants felt their anxiety increased their receptivity to a conversation about physical activity because of the relevance of discussing cancer risk-reducing behaviours at that time, as previously discussed.

I think maybe at the time of the appointment is probably best… while you’re still a bit nervous… You know, ‘cause after it’s done like I’m on to the next thing. (Cervical screening, Female, #26)

Participants who felt they would not have been open to a discussion during the screening appointment thought they would be more receptive at an alternative time, such as after receiving their results.

On the actual day it is taboo, as far as I'm concerned, but once you get your results…you think…what have I been doing that helps me to avoid it… you could build in… a letter or a follow-up phone call. And just say, *‘*well, you've had all your results…; would you be receptive to having a discussion.’ (Breast screening, Female, #15)

One participant also felt that if someone had tried to speak to her about physical activity during screening, this would have caused her anxiety about her screening result because it would have suggested to her that something concerning had been seen during the examination.

‘If you’ve done the test, and then you sit down with someone and they’re like… ‘let’s talk about how we can reduce cancer.’ You’re literally going to go, well, what have you seen? (Cervical screening, Female, #30)

### Communication styles of healthcare professionals

For some participants, the perceived main determinant of receptivity was the communication style of healthcare professionals rather than how they felt during cancer screening. These participants felt their openness to a conversation would depend on how physical activity would be discussed by healthcare professionals.

I think it would depend on the person that was doing it. Because I’ve had three people that have struck me as quite different styles and different people. (Breast screening, Female, #3).

Participants with this view felt if information was delivered in a judgemental way, it could have negative consequences for future screening participation.

I think it depends how it’s done, and how they say it, and, and whether or not you’re doing the right thing or not. I think, nobody likes to be… told off, do they? (Breast screening, Female, #20)

A participant stated that they would be deterred from attending cancer screening if they felt they would be told off about their current behaviour.

I think it probably isn’t the right time, because I think it’s more likely that it would put people off… it might put me off from going, if I knew, if somebody was gonna… have a go at me*.* (Breast screening, Female, #20)

Participants also explained that the characteristics and expertise of the healthcare professional who facilitated potential discussions were important to them. Participants wanted a discussion to be with someone who would be able to answer questions about physical activity, particularly specific questions about how the advice related to them.

Somebody who you can actually ask… a medical question and they can answer it (Cervical screening, Female, #1).

Participants felt they already knew that being physically active reduced their risk of developing cancer and would prefer to receive personalised advice about cancer risk reduction.

I’m like, “Yes, yes. I know this…Yes, exercise good. Yes, I am aware of this”…So I think if it’s like tailored to the individual then that’s good (Cervical screening, Female, #4).

## Discussion

In this study of UK participants who had recently attended a cancer screening appointment, receptivity to a proposed physical activity discussion was influenced by participants’ affective response to screening as well as their views on how and when it would be delivered.

Similar to previous studies, many participants were open to receiving information about physical activity alongside cancer screening and felt it was a convenient time for physical activity to be discussed.^19 27^ Some participants also discussed increases in receptivity in response to anxiety about their cancer risk experienced at screening, which is aligned with the teachable moment heuristic.^11^ Conversely, some attendees described feeling highly anxious throughout their screening experience which could be related to the screening examination or their concerns about their risk of cancer. Some participants felt this anxiety would reduce their receptivity and ability to engage in a discussion about physical activity. This is in line with concerns reported by physicians within a lung cancer setting who highlighted the potential for screening participants to be too overwhelmed to take in information about health behaviours.^28^ For these attendees, it may be beneficial to discuss cancer prevention advice after results have been received, when their anxiety has reduced. Previous studies have suggested around 30% of people who are open to receiving behaviour change advice want it at a time other than their screening appointment.^19^ To ensure all screening attendees receive physical activity advice at a time appropriate for them, discussions could be offered both during and after the results of screening.

The communication style of healthcare professionals was also key to some participants’ receptivity. In line with qualitative studies exploring the acceptability of alcohol reduction and smoking cessation advice in screening contexts, participants wanted advice that was non-judgemental.^12 17^ Non-judgemental communication could include avoiding a tone or language that may be considered criticising or stigmatising. Instead, a sensitive, personalised approach that takes into account participants’ wider contexts and preferences may be better received and reduce the risk of deterring people from future screening attendance. Screening attendees may also find it helpful if context to physical activity discussions is given, for example, explaining that cancer prevention advice is being offered to all attendees opportunistically. Otherwise, attendees may get concerned that an abnormality has been detected. Although a personalised approach fits with the NHS Long Term Plan, healthcare professionals may also need further training in exercise medicine to be able to provide the tailored information screening attendees seek.^29^

It is currently unknown whether conversations about physical activity delivered during cancer screening would be effective at helping patients increase their physical activity level. It is also not known whether delivering conversations about physical activity alongside cancer screening could still deter people from future screening, even if the interventions were delivered in a way that is acceptable to most patients. The risks and ethics of deterring people from screening need to be carefully considered before interventions are trialled.

This study is the first to our knowledge to qualitatively explore cancer screening attendees’ views about the provision of physical activity advice across multiple NHS cancer screening settings. Patients who had recently had a cancer screening appointment were recruited, which overcomes some of the limitations of considering hypothetical scenarios in previous studies.^19 27^ Furthermore, the use of DPIs meant participants were able to reflect on how open they actually felt at the time as opposed to relying on recall or asking how they might feel at future appointments.

Several limitations of this research need to be acknowledged. First, participants were still asked about their receptivity to a hypothetical discussion, and attendees may respond differently if the discussions took place. Second, although the study was designed to maximise information power, through a clearly defined aim, a specific and relevant sample, and the use of DPIs that generated rich, reflective accounts, its full achievement may have been constrained by the composition of the sample.^30^ Most participants were female and cervical screening attendees, and only three were from the NHS bowel screening programme, limiting the breadth of perspectives we captured. Views of participants from breast, cancer and bowel cancer screening were considered together, but it is possible that there are differences in openness to a physical activity intervention between genders and the screening programmes, which could be explored in future research. Additionally, the methods used to identify screening participants may have resulted in a sample who hold more positive views about participating in cancer screening and/or physical activity. Two thirds of the sample were educated to degree level or above. Higher levels of education are associated with greater levels of willingness to receive information about physical activity alongside cancer screening.^19^ It is also possible that the participants in ‘Conversation Time’ who were more interested in discussing this topic volunteered to be interviewed. However, those who completed ‘Conversation Time’ but were not interviewed had similar characteristics to the interview sample, for example, the majority were female and cervical cancer attendees. The ages, ethnicities and education levels of the two groups were also similar. This study was not designed to investigate associations, but it is possible that participant sociodemographics and screening experience are associated with openness. Previous survey research looking at determinants of openness to advice about PA at cancer screening suggested potential differences according to ethnicity, but no other sociodemographic determinants.^27^ In previous research, cancer risk factor awareness was also associated with openness to advice about PA, but the authors did not ask about screening experience, which could be explored in future studies.^27^ The focus of this study was physical activity. However, advice about other important cancer risk factors, such as smoking, alcohol consumption, body weight or diet, may be received differently by screening participants. Finally, the researchers believe the NHS cancer screening programmes are an opportunity to discuss physical activity. Although the researchers attempted to remain neutral throughout the study, it is possible these views influenced how the data were gathered and interpreted.

## Conclusions

Cancer screening attendees’ receptivity to discussing physical activity could be influenced by the timing of a discussion and by their levels of anxiety throughout screening. For some participants, their anxiety during screening reduces their ability to engage in a discussion, but for others, it increases the relevance of the discussion. The way physical activity is discussed was key for why some screening attendees could be deterred from attending future cancer screening.

## Supplementary material

10.1136/bmjopen-2025-099416 online supplemental file 1

## Data Availability

No data are available.
