# Supplementary material for: ‘I think it depends how it’s done’: a qualitative study of screening attendees’ perspectives on receiving physical activity advice within UK NHS cancer screening programmes
Source: BMJ Open. 2025 Nov 21;15(11):e099416. doi: 10.1136/bmjopen-2025-099416 (PMC12645609; doi:10.1136/bmjopen-2025-099416)
Supplement: online supplemental file 1 [file bmjopen-15-11-s001.docx]

1. Data-prompted interview topic guide

**Part 2: Openness to a conversation about physical activity and cancer prevention**

“The second part of this interview follows on from the final graph that we looked at, about your openness to a conversation about physical activity and cancer prevention”

If participant **is open** to a conversation about PA and cancer prevention

1. I see that you had a really high score for openness for this type of discussion on day xx. Could you tell me more about why you think you felt that way then?

Or/And

1. I see that you weren’t so open to this kind of discussion on day xx – could you tell me more about why that was? What was different between this day and the other day [the day that they had a high score for openness]?

Where necessary and appropriate, the following prompts will be used.

1. When do you think would be the best time to have a conversation about physical activity and cancer prevention?
2. Why do you think that this would be the best time to have that conversation?
3. What kind of information would you like to receive in this conversation? Why?
4. Who do you think would be the best person to have this conversation with? Why?

If participant **is not open** to a conversation about PA and cancer prevention.

1. Why do you think that cancer screening is not the right time to talk about physical activity and cancer prevention?
2. Can you think of a better time to have a conversation about physical activity and cancer prevention? Why?
3. What kind of information would you want to get? Why?
4. Who do you think would be the best person to have this conversation with? Why?
5. Consolidated criteria for reporting qualitative studies (COREQ): 32-item checklist

From: Tong, A., Sainsbury, P., & Craig, J. (2007). Consolidated criteria for reporting qualitative research (COREQ): a 32-item checklist for interviews and focus groups. Int J Qual Health Care, 19(6), 349-357. doi:10.1093/intqhc/mzm042

| **No. Item** | **Guide questions/description** | **Reported in section..** |
| --- | --- | --- |
| **Domain 1: Research team and reﬂexivity** | | |
| *Personal Characteristics* | | |
| 1. Interviewer/facilitator | Which author/s conducted the interview or focus group? | *Procedure* |
| 2. Credentials | What were the researcher’s credentials? E.g. PhD, MD | *Procedure* |
| 3. Occupation | What was their occupation at the time of the study? | *Procedure* |
| 4. Gender | Was the researcher male or female? | *Procedure* |
| 5. Experience and training | What experience or training did the researcher have? | N/A |
| *Relationship with participants* | | |
| 6. Relationship established | Was a relationship established prior to study commencement? | *Procedure* |
| 7. Participant knowledge of the interviewer | What did the participants know about the researcher? e.g. personal goals, reasons for doing the research | *Procedure* |
| 8. Interviewer characteristics | What characteristics were reported about the interviewer/facilitator? e.g. Bias, assumptions, reasons and interests in the research topic | *Discussion* |
| **Domain 2: study design** | | |
| *Theoretical framework* | | |
| 9. Methodological orientation and Theory | What methodological orientation was stated to underpin the study? e.g. grounded theory, discourse analysis, ethnography, phenomenology, content analysis | *Data Analysis* |
| *Participant selection* | | |
| 10. Sampling | How were participants selected? e.g. purposive, convenience, consecutive, snowball | *Recruitment and participants* |
| 11. Method of approach | How were participants approached? e.g. face-to-face, telephone, mail, email | *Recruitment and participants* |
| 12. Sample size | How many participants were in the study? | *Results* |
| 13. Non-participation | How many people refused to participate or dropped out? Reasons? | *Results* |
| *Setting* | | |
| 14. Setting of data collection | Where was the data collected? e.g. home, clinic, workplace | *Procedure* |
| 15. Presence of non-participants | Was anyone else present besides the participants and researchers? | *Procedure* |
| 16. Description of sample | What are the important characteristics of the sample? e.g. demographic data, date | *Results* |
| *Data collection* | | |
| 17. Interview guide | Were questions, prompts, guides provided by the authors? Was it pilot tested? | *Procedure* |
| 18. Repeat interviews | Were repeat interviews carried out? If yes, how many? | N/A |
| 19. Audio/visual recording | Did the research use audio or visual recording to collect the data? | *Procedure* |
| 20. Field notes | Were ﬁeld notes made during and/or after the interview or focus group? | *Data analysis* |
| 21. Duration | What was the duration of the inter views or focus group? | *Procedure* |
| 22. Data saturation | Was data saturation discussed? | *Data analysis* |
| 23. Transcripts returned | Were transcripts returned to participants for comment and/or correction? | N/A |
| **Domain 3: analysis and ﬁndings** | | |
| *Data analysis* | | |
| 24. Number of data coders | How many data coders coded the data? | *Data analysis* |
| 25. Description of the coding tree | Did authors provide a description of the coding tree? | *Appendix C* |
| 26. Derivation of themes | Were themes identiﬁed in advance or derived from the data? | *Data analysis* |
| 27. Software | What software, if applicable, was used to manage the data? | *Data analysis* |
| 28. Participant checking | Did participants provide feedback on the ﬁndings? | N/A |
| *Reporting* | | |
| 29. Quotations presented | Were participant quotations presented to illustrate the themes/ﬁndings? Was each quotation identiﬁed? e.g. participant number | *Results* |
| 30. Data and ﬁndings consistent | Was there consistency between the data presented and the ﬁndings? | *Results* |
| 31. Clarity of major themes | Were major themes clearly presented in the ﬁndings? | *Results* |
| 32. Clarity of minor themes | Is there a description of diverse cases or discussion of minor themes? | *Results* |

1. Example of analytic framework

| **Receptivity to physical activity advice (example)** | | | | | | | | |
| --- | --- | --- | --- | --- | --- | --- | --- | --- |
| **ID** | **Previous experience of discussing behaviour change with healthcare professionals** | **Information needs and preferences** | **General receptivity to PA advice** | **Receptivity to PA advice at cancer screening** | **Screening as a teachable moment** | **Timeliness of the teachable moment** | **Information overload** | **Information in relation to screening results** |
| 11 | N/A | Participant would like information about what is going on locally e.g. exercise classes (p12)  Important for information to be linked to the doctors surgery and to come from a healthcare professional (p13).  “I’d rather have that conversation with a professional in an appointment instead of going online to scare myself.” (p14) | This person’s general receptivity (not @ screening appointment specifically) was highly dependent on what else is going on at that time (e.g. work and other commitments) (p11). | Participant felt that screening was a good opportunity to discuss PA and cancer prevention  Participant thought that they would have felt comfortable having that conversation as they had already exposed themselves (p14).  “Yeah. You’ve already just exposed your, umm, [laughing] yeah…There’s no, there’s no conversation you can’t broach at that point.” (p14) | Participant thought that as cancer is already going to be playing on people’s minds, cancer screening would be a good opportunity to give information (p13) | Participant was highly anxious in the lead up to the screening appointment, but some of the stress did go away after the appointment (p9). |  |  |
