# Supplementary material for: ‘I think it depends how it’s done’: a qualitative study of screening attendees’ perspectives on receiving physical activity advice within UK NHS cancer screening programmes
Source: BMJ Open. 2025 Nov 21;15(11):e099416. doi: 10.1136/bmjopen-2025-099416 (PMC12645609; doi:10.1136/bmjopen-2025-099416)
Supplement: Supplementary file 2 [file bmjopen-2025-099416.reviewer_comments.pdf]

PEER REVIEW HISTORY

BMJ Open publishes all reviews undertaken for accepted manuscripts. Reviewers are asked to complete a checklist review form and are provided with free text boxes to elaborate on their assessment. These free text comments are reproduced below.

ARTICLE DETAILS

Title (Provisional)

“I think it depends how it’s done”: a qualitative study of screening attendees’ perspectives on receiving physical activity advice within UK NHS cancer screening programmes

Authors

Murphy, James; Stevens, Claire; Roberts, Anna L; Vrinten, Charlotte; Waller, Jo; Smith, Samuel; Beeken, Rebecca J

VERSION 1 - REVIEW

**Reviewer**	**1**
**Name**	**Wilson, Oliver**
**Affiliation**	**National Institute on Minority Health and Health Disparities (NIMHD**
**Date**	**06-Mar-2025**
**COI**	**None**

This is a well-intentioned study on an important topic. However, the level of scientific writing and attention to detail was relatively poor. The qualitative methodology adopted is unclear, and the way in which qualitative findings has been reported makes it impossible for readers to properly interpret findings. Regrettably, these shortcomings made reviewing the Results and Discussion incredibly difficult.

Abstract

Generally sentences are not started with numerals, but rather with numbers written out in word form. The authors may want to consider rewording or rearranging content within the abstract to avoid this

Data ‘prompted’ is used in the Abstract, but ‘promoted’ is used in the Strengths. Which one is it? (note, this is not consistent in the manuscript either)

The first Limitations bullet point does not make sense grammatically

Pooling qualitative data is an unusual way to describe things, I would suggest reconsidering this wording in the second Limitations bullet

Background

Please define what 'being physically active' is the first paragraph. Also, please reconsider specifying what 'this' in 'increase this' is

If the aim was to 'explore variations', adopting a qualitative approach would not be advisable. I suggest the authors reframe their aims, and align them with what their chosen research approach is capable of actually exploring

Methods

Please explain what 'Call for Participants' is

Please offer more details on the inclusion/exclusion criteria. Was the study conducted in a specific geographic location, when does screening for cancer start in the UK, etc.?

How were participants compensated?

What is the "The framework method of data Management". The citation offered is not helpful, and I have never come across this method of qualitative analysis before. Themes are not even mentioned within the methods, yet suddenly appear in the Results. It appears that a basic inductive thematic analysis has been attempted. However, the methods and results do not reflect this. Once the findings have been weighted appropriately, the discussion and conclusions of this study may change

Results

The researchers had 30 participants, so some details should be being offered to readers as to how many of these participants made comments regarding the 'themes' that the researchers have come up with. At present, readers have no idea how much weight to give to each theme. Anywhere from 2 to 30 participants could have made a comment relating to a given theme. At present, the authors just say participants throughout, so there is a risk in someone interpreting the findings as if all participants made comments on each theme (which is not possible). Information on the number of participants making a comment relating to each theme needs to be reported to establish the weight a reader should give to each theme

Every single quote is from a female, who were over represented. Did anything at all stand out about the male responses?

Discussion

It is not possible for me to offer comments on the Discussion since the Results, as they have been reported, cannot be interpreted.

**Reviewer**	**2**
**Name**	**Sarkeala, Tytti**

**Affiliation**	**Finnish Cancer Registry, Mass Screening Registry**
**Date**	**19-Mar-2025**
**COI**	**None**

Screening sessions provide appealing settings to reach asymptomatic populations and to discuss about risks factors of cancer. However, additional maneuvers before, during and after screening may influence the willingness to participate and may thus hamper the efficacy of screening. On the other hand, they may also provide a teachable moment to improve participants' lifestyle.

This manuscript assesses possibility to integrate physical activity advice within cancer screening. The data are driven from surveys (5+1+5 by phone) and survey-data-prompted semi-structured interviews. The results show variability in opinions and reactions and underline the importance of communication style.

Comments on study design:

- I did not find information on channels through which the participants of the study were eventually recruited, please provide.

- How did you get information on the upcoming screening appointment? Why did you think it was important that the appointment was within two weeks of joining the study?

- The survey took eleven days, five times per day

o Why did you choose this kind of an exhaustive approach?

o How many persons dropped out of the survey (i.e. started but did not finish it)?

o How did those starting and quitting or completing the survey but NOT participating to interviews differ from those agreed to take part also in the interviews?

- Further on selection – education level and gender was mentioned, how about the fact that all respondents were those who completed the 11 day survey AND the interview, and were screening participants?

Other comments:

- How did you address physical activity in the survey and interviews and what were the actual questions concerning the physical activity?

- Based on your results, do you think it is feasible to start informing screening participants on the relationships between physical activity and cancer risk? If yes, why? If not, why?

- Would it be feasible to address, alongside physical activity, also other lifestyle risk factors in the screening appointment, such as alcohol consumption, smoking, nutrition, obesity…

- For curiosity - how would you ascertain a non-judgmental communication style?

**Reviewer**	**3**
**Name**	**Dylke, Elizabeth S**
**Affiliation**	**The University of Sydney**
**Date**	**15-May-2025**
**COI**	**None**

This is a clearly written paper about an interesting and important topic. The rationale and methods for the study are mostly clear and the results are easy to follow. I had a few minor things (below) for specific questions and then some considerations for the discussion and future directions of research from the manuscript.

From the exemplar quotes and participant description in particular, I wondered if there was additional considerations that were important for interpretation of the findings or future research that may be able to be addressed in the discussion. I know that the data from all groups was pooled for the analysis but these ideas jumped out at me from your results.

1) Do you know if the people who volunteered were physically active already? This may have an impact on their comfort and interest with discussing this topic.

2) Is there an impact of age? From the exemplar quotes, it felt as though there may be a difference in perspective between those going for breast cancer screening and those going for cervical cancer screening. These two screening programs have some overlap in the age groups targeted, but the cervical cancer screening group will also have a younger group that may have a different relationship with exercise and physical activity. It would be interesting to explore difference here.

3) Is there an impact of prevalence of the cancer types on anxiety and therefore interest in discussion/timing of discussion on physical activity? Breast cancer is a more common cancer type and many people going through screening will have known people who had cancers detected at screening, increasing their anxiety of a similar outcome. I believe this would be less common in the cervical cancer screening group, who may feel as this is more of a routine procedure. I wonder if this would change their openness and the timing of discussion around physical activity.

Specific questions

Procedure

Pg 5, line 40-41: “ Participants in the overall study were invited to complete a survey 5 times per day, for 11 days (5 days prior to a screening appointment…”

- Did participants have to complete the survey 5 times per day or that you sent them basically 5 reminders per day for those 11 days? This was a bit unclear.

Participant information

- I think it would be helpful to know the range of time after their screening experience that the interviews occurred as this may have impacted their feelings around openness/anxiety etc

- Table 1- as the SD for age is quite wide and possibly not normatively distributed, a median and inter-quartile range might be more appropriate.

VERSION 1 - AUTHOR RESPONSE

<u>Reviewer 1's Comments</u>:

Comment 1: Generally sentences are not started with numerals, but rather with numbers written out in word form. The authors may want to consider rewording or rearranging content within the abstract to avoid this

Response 1: We have reworded multiple sentences to avoid this:

"*Up to 80% of cancer screening attendees are open to discussing physical activity during cancer screening but some say this would deter them from future screening.*" (Abstract, p2)

"*There were 30 participants.*" (Abstract, p2)

Comment 2: Data 'prompted' is used in the Abstract, but 'promoted' is used in the Strengths. Which one is it? (note, this is not consistent in the manuscript either)

Response 2: Thank you for spotting this spelling mistake, it should read 'prompted'. We have corrected this in the Strengths and Limitations section (p3), as well as in the manuscript (p13).

Comment 3: The first Limitations bullet point does not make sense grammatically

Response 3: This bullet point has been edited. It should have read 'or' above rather than 'of'. It now reads:

"*A higher education level is associated with greater acceptability of a physical activity intervention alongside cancer screening. In this sample, two thirds of participants were educated to degree level or above which may have resulted in a sample who held more positive views about receiving information about physical activity than the general population*." (Strengths and Limitations, p3)

Comment 4: Pooling qualitative data is an unusual way to describe things, I would suggest reconsidering this wording in the second Limitations bullet.

Response 4: Thank you for noticing this. We analysed the data together and did not split participants into groups based on the screening programme they attended. We have edited this line to make it clearer.

"*The views of participants were considered collectively. However, it is possible that there were differences in openness to a physical activity intervention between genders and each screening programme*." (Strengths and Limitations, p3)

A corresponding sentence in the manuscript has also been changed. "*Views of participants from breast, cancer and bowel cancer screening were considered together but it is possible that there were differences in openness to a physical activity intervention between genders and the screening programmes.*" (Discussion, p13)

Comment 5: Please define what 'being physically active' is the first paragraph. Also, please reconsider specifying what 'this' in 'increase this' is

Response 5: We would define being physically active as doing regular physical activity to a level that meets UK government / NHS physical activity guidelines. In the final sentences of the opening paragraph 'this' relates to rates of physical activity in the UK. We have edited the opening paragraph.

"*Meeting NHS physical activity guidelines (being physically active) reduces a person's risk of developing multiple types of cancer. However, one third of adults in the UK are not physically active. National guidelines recommend healthcare practitioners discuss physical activity in every patient contact to try to increase rates of physical activity*." (Introduction, p4)

Comment 6: If the aim was to 'explore variations', adopting a qualitative approach would not be advisable. I suggest the authors reframe their aims, and align them with what their chosen research approach is capable of actually exploring.

Response 6: Previous research has shown that the provision of a conversation about physical activity is acceptable to some cancer screening attendees but not others. What is not known is why this is. Understanding the reasons for the variation views is important to aid the development of interventions that could be delivered alongside cancer screening. Qualitative methodology can aid intervention design by helping to develop an in-depth understanding of patient experience and preferences (Yardley, Morrison, Bradbury, & Muller 2015). We have edited this section and a corresponding sentence in the abstract.

"*Therefore, the aims of this study were to gain an in-depth understanding of individual differences in receptivity to physical activity advice at cancer screening*" (Introduction, p4)

Reference:

Yardley, L., Morrison, L., Bradbury, K., & Muller, I. (2015). The Person-Based Approach to Intervention Development: Application to Digital Health-Related Behavior Change Interventions. *Journal of Medical Internet Research, 17*(1). doi:10.2196/jmir.4055

Comment 7: Please explain what 'Call for Participants' is

Response 7: The 'Call for Participants' is a recruitment website where researchers can advertise their studies. We placed an advert on this website to aid our recruitment. The website can be found at https://www.callforparticipants.com. We have edited the beginning of the Recruitment and Participants section. It now reads:

"For 'Conversation Time' participants were recruited by adverts posted on Facebook and the 'Call for Participants' website, and posters were placed around two university campuses." (Recruitment and Participants p5)

Comment 8: Please offer more details on the inclusion/exclusion criteria. Was the study conducted in a specific geographic location, when does screening for cancer start in the UK, etc.?

Response 8: Information about the inclusion/exclusion criteria for both 'Conversation Time' and this study are included in the methods section. There were no further criteria used in either study. We have now added details about the location into the title, abstract and methods sections based on comments from the editor and yourself (please see Editor comment and response 1).

Comment 9: How were participants compensated?

Response 9: Participants recruited from university campuses or online were given a £20 shopping voucher. Participants recruited from the participant recruitment agency were given £25, as this was the charge set by the recruitment agency. We have added a line to the manuscript to acknowledge this.

"*Participants were given a small monetary gift as an acknowledgement of the time taken to participate in this research*." (Recruitment and participants, p6)

Comment 10: What is the "The framework method of data Management". The citation offered is not helpful, and I have never come across this method of qualitative analysis before. Themes are not even mentioned within the methods, yet suddenly appear in the Results. It appears that a basic inductive thematic analysis has been attempted. However, the methods and results do not reflect this. Once the findings have been weighted appropriately, the discussion and conclusions of this study may change.

Response 10: The framework method is a common approach used in the management and analysis of qualitative data. The framework method is used to organise and categorise qualitative data and enable comparisons between participants. We used the framework method to manage the data and then performed a thematic analysis. We have edited a sentence in the methods to state this.

"*The framework method of data management was used and a thematic analysis performed*." (Data analysis, p7)

We have outlined the steps we took in our analysis including themes in the methods section. We have also included an additional reference (Gale et al. 2013) regarding framework methodology that may be helpful for readers.

Reference:

Gale K, Heath G, Cameron E. et al. Using the framework method for the analysis of qualitative data in multi-disciplinary health research. BMC Med Res Methodol 13, 117 (2013). https://doi.org/10.1186/1471-2288-13-117

Comment 11: The researchers had 30 participants, so some details should be being offered to readers as to how many of these participants made comments regarding the 'themes' that the researchers have come up with. At present, readers have no idea how much weight to give to each theme. Anywhere from 2 to 30 participants could have made a comment relating to a given theme. At present, the authors just say participants throughout, so there is a risk in someone interpreting the findings as if all participants made comments on each theme (which is not possible). Information on the number of participants making a comment relating to each theme needs to be reported to establish the weight a reader should give to each theme.

Response 11: Thank you for this comment. As a research team we have discussed reporting theme frequency on multiple occasions. There are differing views in qualitative research, but prominent

researchers are "critical" of the reporting of theme frequency (Braun and Clarke 2020, p336). We feel that as our aim was to explore individual differences in acceptability, the frequency of each theme is not beneficial to our research question. Additionally, we feel that by providing quantitative values alongside the qualitative data that undue weight could be given to particular themes when their value should be based on the theme's significance in answering the research question.

Reference: Braun V & Clarke V. 2020. One size fits all? What counts as quality practice in (reflexive) thematic analysis? Qualitative Research in Psychology, 18(3), 328–352. https://doi.org/10.1080/14780887.2020.1769238

Comment 12: Every single quote is from a female, who were over represented. Did anything at all stand out about the male responses?

Response 12: The sample was mostly female which reflects there are more NHS screening programmes available to women compared with men. We recruited recent cancer screening attendees so it is not unsurprising that our sample was mostly female. The sample characteristics are also similar to a quantitative on this topic, in which males accounted for 14% of the sample (Stevens et al. 2019). In our study we did not feel there were any prominent themes discussed specifically by the male participants. We have chosen to present what we felt were the most prominent themes as well as the quotes we feel best provide evidence for them. We do acknowledge that the views of males are under-represented and that this is a limitation of this study. We have already included a line about this in the discussion section.

Reference:

Stevens C, Vrinten C, Smith SG. et al. Acceptability of receiving lifestyle advice at cervical, breast and bowel cancer screening. Prev Med. 2019;120:19-25. doi:10.1016/j.ypmed.2018.12.005

Comment 13: It is not possible for me to offer comments on the Discussion since the Results, as they have been reported, cannot be interpreted.

Response 13: We politely disagree with this response. The results have been analysed in line with established qualitative approaches and prepared according to the consolidated criteria for reporting qualitative research checklist. We are unable to respond to this query without further clarification on what cannot be interpreted. We hope that the changes to the manuscript and additional information we have provided has been helpful and we are happy to respond to additional specific queries.

<u>Reviewer 2's Comments</u>:

Comment 1: I did not find information on channels through which the participants of the study were eventually recruited, please provide.

Response 1: We have added more detail to the results section to provide more information on this.

"*Of the 41 participants involved in 'Conversation Time', 30 agreed to take part in this study. Of the 30 participants 4 had been recruited via the adverts placed online and on university campuses and 26 were recruited via the specialist participant recruitment agency*." (Sample characteristics, p8)

Comment 2: How did you get information on the upcoming screening appointment? Why did you think it was important that the appointment was within two weeks of joining the study?

Response 2: Participants for 'Conversation Time' were recruited from the general population in England. The key eligibility criteria was that they had an upcoming cancer screening appointment. The time frame was set at 2 weeks because the aim was to recruit participants with upcoming cancer screening so that their experience could be explored soon after whilst it was recent. In 'Conversation Time' it was important that there was some time prior to the screening appointment to allow for the consent process, app enrolment and five days of measures prior to the cancer screening appointment. We recruited recent cancer screening attendees to improve on previous research where this subject was broached in a hypothetical discussions as mentioned in the discussion. We have added the following sentence to the methods:

"This time limit was set to include participants who had recent experience of cancer screening and to allow sufficient time for the study procedures prior to the cancer screening appointment."
(Recruitment and participants, p6)

Comment 3: The survey took eleven days, five times per day - Why did you choose this kind of an exhaustive approach?

Response 3: The surveys were a form of Ecological Momentary Assessment (EMA) which is a method of repeatedly capturing participants behaviour, in this case their openness to having a conversation about physical activity, in real-time. It is common in EMA to collect data five to six times a day from participants. This provides rich data about participant's behaviour and can demonstrate changes over time. In our study participants were provided with a copy of their EMA data during the interview because this can help prompt their memory of how they felt at certain points and how their feelings changed over time. This was particularly important for this study, which sought to understand differences in participants receptivity around the time of the cancer screening appointment. It was anticipated from previous work by our research group that not all participants would be open to lifestyle advice at cancer screening. Therefore, the combination of EMA and the data-prompted interviews were selected to explore the differing levels of openness to physical activity advice over time. A paragraph has been added to the methods section to discuss this in the manuscript (p6).

"*The 'Conversation Time' study used Ecological Momentary Assessment (EMA) which is a method of capturing repeated measurements of behaviour in real-time*". (Recruitment and participants, p5)

"Providing participants with EMA data enables data prompted interviews (DPIs) in which these data can guide discussion and help participants understand and articulate processes which may change over time. DPIs using EMA data can help researchers to understand between-person variation in an outcome in line with our research question." (Procedure, p6)

Comment 4: How many persons dropped out of the survey (i.e. started but did not finish it)?

Response 4: For 'Conversation Time' 49 participants consented and 41 participated in the study. All 41 participants who participated in the study completed the study. Of the 41 participants in 'Conversation Time' 30 participants took part in this study. This information was not included in the paper but could be added if it was thought to be useful.

Comment 5: How did those starting and quitting or completing the survey but NOT participating to interviews differ from those agreed to take part also in the interviews?

Response 5: The participants who did not complete 'Conversation Time' and those that did not participate in this study were similar in their baseline characteristics to the sample. In the group who didn't participate in the interviews, every person was female and just over half had attended cervical

cancer screening (54%). The ages, ethnicities and education levels of the two groups were also similar. This information was not included in the paper but could be added if it is helpful.

Comment 6: Further on selection – education level and gender was mentioned, how about the fact that all respondents were those who completed the 11-day survey AND the interview, and were screening participants?

Response 6: We thank the reviewer for this comment and agree self-selection bias could have occurred from the convenience sampling approach in this study. It is possible that the participants in 'Conversation Time' who were more interested in discussing this topic or felt more strongly about their viewpoint volunteered to be involved in this study. This may have impacted our results and may reduce their transferability. We have added these sentences in to the discussion to illustrate this.

"It is also possible that the participants in 'Conversation Time' who were more interested in discussing this topic volunteered to be involved in this study. This may have impacted our results and may reduce their transferability." (Discussion, p12)

Comment 7: How did you address physical activity in the survey and interviews and what were the actual questions concerning the physical activity?

Response 7: In 'Conversation Time' participants were asked the exercise vital sign (Kuntz et al. 2021). The topic guide used in the interventions is included in Appendix A. Participants were asked why they were or were not open to a conversation about physical activity and their reasons for this were then explored. Other questions related to the timing, content and delivery of a conversation about physical activity. We have not included additional information about 'Conversation Time' but we have edited a sentence in the methods to in relation to this.

"A semi-structured interview topic guide was created. In the second part of this participants were asked about their openness to having a conversation about physical activity at their cancer screening appointment (Appendix A)." (Procedure, p6)

Reference:

Kuntz J, Young D, Saelens B et al. Validity of the Exercise Vital Sign Tool to Assess Physical Activity. Am J Prev Med. 2021 Jun;60(6):866-872. doi: 10.1016/j.amepre.2021.01.012. Epub 2021 Mar 27. PMID: 33781618; PMCID: PMC8154650.

Comment 8: Based on your results, do you think it is feasible to start informing screening participants on the relationships between physical activity and cancer risk? If yes, why? If not, why?

Response 8: This study aimed to explore reasons for differences in participants receptivity to a conversation about physical activity. Although many participants were open to a conversation there were some participants that felt they could be put off attending screening by healthcare professionals discussing physical activity. The results of this study aid researchers' understanding of the differences of cancer screening attendees' receptivity. However, it is not known whether physical activity interventions designed even with this knowledge will be acceptable to cancer screening attendees and not deter them. Furthermore, the effectiveness and feasibility of delivering an intervention acceptable to patients has not been explored in this study. This would require further research to investigate.

"It is currently unknown whether conversations about physical activity delivered during cancer screening would be effective at helping patients increase their physical activity level. It is also not

known whether delivering conversations about physical activity alongside cancer screening could still deter people from future screening, even if the interventions were delivered in a way that is acceptable to most patients. The risks and ethics of deterring people from screening needs to be carefully considered before interventions are trialled." (Discussion, p12)

Comment 9: Would it be feasible to address, alongside physical activity, also other lifestyle risk factors in the screening appointment, such as alcohol consumption, smoking, nutrition, obesity…

Response 9: There is evidence that addressing other lifestyle risk factors such as alcohol consumption, smoking, diet and obesity alongside cancer is acceptable to patients (See Chambers et al. 2019 & Sinclair et al. 2020) as well as effective (See Moldovanu et al. 2021 & Orange et al. 2021). The focus of this paper was physical activity so discussion about other risk factors was not included in the manuscript to keep it concise.

References:

Chambers SE, Copson ER, Dutey-Magni PF et al. Alcohol use and breast cancer risk: A qualitative study of women's perspectives to inform the development of a preventative intervention in breast clinics. Eur J Cancer Care (Engl). 2019;28(4):e13075. doi:10.1111/ecc.13075

Moldovanu D, de Koning HJ, van der Aalst CM. Lung cancer screening and smoking cessation efforts. Transl Lung Cancer Res. 2021;10(2):1099-1109. doi:10.21037/tlcr-20-899

Orange S, Hicks K & Saxton J. Effectiveness of diet and physical activity interventions amongst adults attending colorectal and breast cancer screening: a systematic review and meta-analysis. Cancer Causes Control 32, 13–26 (2021). https://doi.org/10.1007/s10552-020-01362-5

Sinclair J, Dutey-Magni P, Anderson A et al. A Context-Specific Digital Alcohol Brief Intervention in Symptomatic Breast Clinics (Abreast of Health): Development and Usability Study. JMIR Res Protoc 2020;9(1):e14580. DOI: 10.2196/14580.

Comment 10: For curiosity - how would you ascertain a non-judgmental communication style?

Response 10: To communicate in a non-judgemental way, one should avoid a tone or language that may be considered criticising or stigmatising. A key fundamental to most communication is good listening, and this is also important if communication is to be perceived as non-judgemental. There are different communication styles that are thought to be non-judgemental in nature. One such style is motivational interviewing which can be used to help people change their behaviour and is non-judgemental in spirit (See Miller and Rollnick 2013 & O'Halloran et al. 2014). We have added a line into the text in relation to this.

"Non-judgemental communication could include avoiding a tone or language that may be considered criticising or stigmatising." (Discussion, p12).

References

Miller W and Rollnick S. Motivational interviewing: Helping people change. 3rd ed. New York: The Guilford Press, 2013.

O'Halloran PD, Blackstock F, Shields N et al. Motivational interviewing to increase physical activity in people with chronic health conditions: a systematic review and meta-analysis. Clin Rehabil. 2014 Dec;28(12):1159-71. doi: 10.1177/0269215514536210. Epub 2014 Jun 18. PMID: 24942478.

Reviewer 3's Comments:

Comment 1: Do you know if the people who volunteered were physically active already? This may have an impact on their comfort and interest with discussing this topic.

Response 1: We thank the reviewer for this comment. The physical activity levels of participants were assessed in 'Conversation Time'. The physical activity levels varied across the 'Conversation Time' sample (Mean days of exercise per week, 2.82 (SD 2.13), average number of mins per episode 37.31 (SD 24.62)). Participants did not discuss their current exercise habits in relation to their openness to receiving advice. It is also possible that participants who were more interested in physical activity volunteered to take part in the study. There is a sentence in the discussion in relation to this.

"It is also possible that the participants who volunteered for the study were already interested in physical activity and so could have expressed more willingness to discuss physical activity. This may have impacted our results and may reduce their transferability." (Discussion, p13)

Comment 2: Is there an impact of age? From the exemplar quotes, it felt as though there may be a difference in perspective between those going for breast cancer screening and those going for cervical cancer screening. These two screening programs have some overlap in the age groups targeted, but the cervical cancer screening group will also have a younger group that may have a different relationship with exercise and physical activity. It would be interesting to explore difference here.

Response 2: We thank the reviewer for this comment. We agree this would be interesting to explore. This study was not designed to look at associations and we would not be able to say this for definite. We agree there may be differences between the screening programmes and this could relate to several factors including age. This is something that could be investigated in the future. We have added a line to the discussion relating to this.

"This study was not designed to investigate associations, but it is possible that participant sociodemographics and screening experience are associated with openness. Previous survey research looking at determinants of openness to advice about PA at cancer screening suggested potential differences according to ethnicity, but no other sociodemographic determinants.[23] Cancer risk factor awareness was also associated, but the study did not ask about screening experience or look at differences between screening modalities, which could be explored in future research." (Discussion, p13).

Comment 3: Is there an impact of prevalence of the cancer types on anxiety and therefore interest in discussion/timing of discussion on physical activity? Breast cancer is a more common cancer type and many people going through screening will have known people who had cancers detected at screening, increasing their anxiety of a similar outcome. I believe this would be less common in the cervical cancer screening group, who may feel as this is more of a routine procedure. I wonder if this would change their openness and the timing of discussion around physical activity.

Response 3: We did not collect data that would be able to answer the question regarding the impact of prevalence on anxiety and subsequent interest in physical activity, but we agree it's a useful idea that could be explored in future work. We have added the following line to the discussion (p13) following your comment.

"This study was not designed to investigate associations, but it is possible that participant sociodemographics and screening experience are associated with openness. Previous survey research looking at determinants of openness to advice about PA at cancer screening suggested potential differences according to ethnicity, but no other sociodemographic determinants.[23] Cancer risk factor awareness was also associated, but the study did not ask about screening experience or look at differences between screening modalities, which could be explored in future research." (Discussion, p13).

Comment 4: Procedure - Pg 5, line 40-41: "Participants in the overall study were invited to complete a survey 5 times per day, for 11 days (5 days prior to a screening appointment…" - Did participants have to complete the survey 5 times per day or that you sent them basically 5 reminders per day for those 11 days? This was a bit unclear.

Response 4: Participants in the 'Conversation Time' study were invited to complete the survey 5 times per day. The response rate was 82.7% with a range of 14.6% - 100%. This has not been included in the manuscript because we did not feel this was relevant to this study as participants were invited to take part regardless of their completion rate. We have not changed this sentence in the manuscript because of this.

"*Participants in 'Conversation Time' were invited to complete a survey 5 times per day, for 11 days (5 days prior to a screening appointment, on the day of screening, and the 5 days post appointment) using a mobile phone application.*" (Recruitment and participants, p6)

Comment 5: Participant information - I think it would be helpful to know the range of time after their screening experience that the interviews occurred as this may have impacted their feelings around openness/anxiety etc

Response 5: We thank the reviewer for this comment. All the interviews occurred within 3 weeks of the participants cancer screening appointment. There was no indication during the interviews that the views of the participants had changed since the interviews. The interviews were also data prompted which can aid participants re-call and guide their reflection of their feelings at the time of screening. We have added this additional information to the methods section.

"Interviews were conducted within three weeks of the participant's cancer screening appointment." (Procedure, p6)

Comment 6: Table 1- as the SD for age is quite wide and possibly not normatively distributed, a median and inter-quartile range might be more appropriate.

Response 6: We thank the reviewer for this comment. We felt the range (27-69 years) might provide more information to the reader so we have provided that. We have added this information to Table 1.

	Mean (range)
**Age**	44.4 (27-69 years)

VERSION 2 - REVIEW

**Reviewer**	**1**
**Name**	**Wilson, Oliver**
**Affiliation**	**National Institute on Minority Health and Health Disparities (NIMHD**
**Date**	**12-Sep-2025**
**COI**	

This is a fantastic paper, and the authors have done a solid job of responding to comments. All of comments below are relatively minor, and could arguably be considered in the realm of personal preferences. I've tried to keep them high level, and offer them as things for the authors to consider as they make final revisions for a paper that is hopefully accepted.

Stating the guidelines is helpful, but what level of physical activity (in terms of minutes or days/week) is considered to be 'physically active'. Physically activity researchers may know, but your wider audience who is less familiar with guidelines may not

The revised aim is an improvement. 'In-depth' helps shift the aim away from something that could be evaluated quantitively, but the authors could consider going further. In your response, the citation supports 'experiences and preferences', but this framing is missing from the actual text. Including this framing, or something pertaining to the WHY of why differences exist, would convince me as a reader that the right approach was adopted to answer the research question. At present, it still reads a more focused on what differences are. I don't feel super strongly about this, I defer to the authors to decide further edits are warranted

Consider including the actual compensation in the paper unless there is a reason against it. Many reporting checklists encourage the reporting of such details. I understand you may not want to disclose different levels of compensation for different groups, so you could just go with the lower amount and you would head of any questions you may get from someone in the future

Regarding theme frequency, I suggest the authors explain and justify their approach in their methods. It is helpful to explain to readers why decisions like this have been made, as it helps orient them to how to interpret the findings you are reporting? To me, reporting frequency is helpful as it allows the reader to decide how to weight results. By not reporting the frequency, you're arguably weighting the Results in a different way. Both ways are justified, but the approach should be explained/justified.

Reviewer comments can be incredibly annoying at times, and sometimes totally off topic. However, it is important to keep in mind that their reviewer queries are likely reflective of questions that eventual readers are likely to ask, and to take the feedback on board to integrate responses into text when possible. In response to several comments to reviewers,

the authors have chosen to explain things to the reviewer, but not the manuscript. This is probably implied, but for transparency I am Reviewer 1. An example would be the response to Reviewer 2's comment number 9. The authors response is fantastic, but has not been translated into the text. It would require a sentence, but I think it is doable and would be helpful to future readers. Reviewer 2's Comment 5 is another example, it's a fair question that has been answered to the reviewer. I'm on a similar page to you, as it arguably a more niche question, but taking the path of least resistance by proactively answering the question rather than responding "This information was not included in the paper but could be added if it is helpful." Word limits are always a pain, but in multiple instances the authors may have been too hasty to decide to answer comments directly to reviewers as opposed to reviewers and future readers. Perhaps the authors could keep this in mind as they make their final touches

---

**Reviewer**	**3**
**Name**	**Dylke, Elizabeth S**
**Affiliation**	**The University of Sydney**
**Date**	**30-Sep-2025**
**COI**	

Thank you for making a number of changes in responses to the reviewers comments. I think there are still a few things that need to be addressed to help the reader be confident in the results of this study.

Strengths and limitations of this study:

- "Patients who had recently had a cancer screening appointment were recruited which overcomes some of the limitations of considering hypothetical scenarios in previous studies."

o The first strength/limitation doesn't really make sense without reading the whole paper and knowing the background challenges- what are the hypothetical scenarios? This is the first strength so should be strong and demonstrate the meaningful contribution this study is contributing to the literature

- "The views of participants were considered collectively. However, it is possible that there were differences in openness to a physical activity intervention between genders and each screening programme."

o I don't really understand the 4th strength/limitation of the study and it doesn't really feel like a strength or limitation of your study. Its not really a finding that you made nor is it a major focus for your discussion (which is the appropriate place for this point to be). The limitation could be that the study did not consider whether there were gender and screening type differences in receptiveness to discussing PA.

Introduction

Pg 4: “Cancer screening programmes in England bring over 5 million people into contact with a healthcare professional each year, this figure was higher before bowel cancer screening moved to faecal occult blood testing.”

- Some punctuation is missing here

Pg 4: “Therefore, the aims of this study were to gain an in-depth understanding of individual differences in receptivity to physical activity advice at cancer screening understand the reasons why physical activity advice could deter participants from cancer screening.”

- I actually don’t feel like this aim aligns well with your results- so either you are not achieving your aim with the results that you are presenting or your aim isn’t right for the data you have. From what you’ve presented as results, it feels like the aim of this study is to explore differences in receptivity to advice about physical activity at the time of cancer screening. The second part of the aim statement as it currently stands makes the study feel biased towards finding out why it wont work and is not really addressed as all that is presented in your results. You even mention in the discussion that its not known if doing this would change people’s engagement with screening

Results

How many participants had indicated in the original survey that they were not open to discussing PA? I think this data should be included in table 1.

Quotes in general

- I know another reviewer raised that all of the quotes are from females and that you felt you had used the best quotes for each section regardless of gender. 12 quotes are used through this results section but they are only from 8 people, with doesn’t feel like its potentially truly representative of the sample. Particularly in the communication section, you have 4 quotes but they are only from 2 people. Also none of the quotes are from people that are undergoing screening for bowel cancer. It would be better to have quotes from more people as well as all cancer screening types to make sure the results are representative of the whole sample.

Conclusion:

Pg 14: “Screening attendees’ receptivity could be influenced by the timing of a discussion and by their levels of anxiety throughout screening”

- This is the first sentence of the conclusion and I think it should be clarified to include PA. E.g. …receptivity to discussing PA….

VERSION 2 - AUTHOR RESPONSE

Reviewer 1's Comments:

Comment 1: Stating the guidelines is helpful, but what level of physical activity (in terms of minutes or days/week) is considered to be 'physically active'. Physically activity researchers may know, but your wider audience who is less familiar with guidelines may not.

Response 1: We thank the reviewer for this comment and have included some further information to make the statement clearer.

"*Meeting NHS physical activity guidelines of at least 150 minutes of moderate intensity physical activity per week (being physically active) reduces a person's risk of developing multiple types of cancer.*[1-5]" (Introduction, p4)

Comment 2: The revised aim is an improvement. 'In-depth' helps shift the aim away from something that could be evaluated quantitively, but the authors could consider going further. In your response, the citation supports 'experiences and preferences', but this framing is missing from the actual text. Including this framing, or something pertaining to the WHY of why differences exist, would convince me as a reader that the right approach was adopted to answer the research question. At present, it still reads a more focused on what differences are. I don't feel super strongly about this, I defer to the authors to decide further edits are warranted.

Response 2: We have edited the aim based on this response and the response to Reviewer 3's comment 4 below.

"*Therefore, the aims of this study were to gain an in-depth understanding of why individual differences in receptivity to physical activity advice exist at cancer screening, and how personal experiences of screening may influence preferences for receiving this advice*." (Introduction, p4)

Comment 3: Consider including the actual compensation in the paper unless there is a reason against it. Many reporting checklists encourage the reporting of such details. I understand you may not want to disclose different levels of compensation for different groups, so you could just go with the lower amount and you would head of any questions you may get from someone in the future.

Response 3: We thank the reviewer for this comment, we have edited the sentence to include an approximation of the compensation the participants received.

"*Participants were given a small monetary gift, of approximately £20, as an acknowledgement of the time taken to participate in this research.*" (Recruitment and participants, p6)

Comment 4: Regarding theme frequency, I suggest the authors explain and justify their approach in their methods. It is helpful to explain to readers why decisions like this have been made, as it helps orient them to how to interpret the findings you are reporting? To me, reporting frequency is helpful as it allows the reader to decide how to weight results. By not reporting the frequency, you're arguably weighting the Results in a different way. Both ways are justified, but the approach should be explained/justified.

Response 4: We thank the reviewer for this comment. We have included the following in the methods section to justify our approach.

“We do not report theme frequency because, it is possible that, by providing quantitative values alongside the qualitative data that undue weight could be given to particular themes when their value should be based on the theme’s significance in answering the research question.[21]” (Procedure, p7)

Reference: Braun V & Clarke V. 2020. One size fits all? What counts as quality practice in (reflexive) thematic analysis? Qualitative Research in Psychology, 18(3), 328–352. https://doi.org/10.1080/14780887.2020.1769238

Comment 5: Reviewer comments can be incredibly annoying at times, and sometimes totally off topic. However, it is important to keep in mind that their reviewer queries are likely reflective of questions that eventual readers are likely to ask, and to take the feedback on board to integrate responses into text when possible. In response to several comments to reviewers, the authors have chosen to explain things to the reviewer, but not the manuscript. This is probably implied, but for transparency I am Reviewer 1. An example would be the response to Reviewer 2’s comment number 9. The authors response is fantastic, but has not been translated into the text. It would require a sentence, but I think it is doable and would be helpful to future readers. Reviewer 2's Comment 5 is another example, it’s a fair question that has been answered to the reviewer. I'm on a similar page to you, as it arguably a more niche question, but taking the path of least resistance by proactively answering the question rather than responding “This information was not included in the paper but could be added if it is helpful.” Word limits are always a pain, but in multiple instances the authors may have been too hasty to decide to answer comments directly to reviewers as opposed to reviewers and future readers. Perhaps the authors could keep this in mind as they make their final touches.

Response 5: We thank the reviewer for this comment which we have taken note of. We have included some of our response to Reviewer 2’s comment 5 in the discussion. We have also included our response to Reviewer 2’s comment 9 in the introduction.

“*It is also possible that the participants in ‘Conversation Time’ who were more interested in discussing this topic volunteered to be interviewed. However, those who completed ‘Conversation Time’ but were not interviewed had similar characteristics to the interview sample, for example, the majority were female and cervical cancer attendees. The ages, ethnicities and education levels of the two groups were also similar.*” (Discussion, p14)

“There is evidence that addressing lifestyle risk factors such as alcohol consumption, smoking, diet and obesity alongside cancer is acceptable to patients.[12-16] However, there is less published research examining the acceptability of physical activity advice alongside cancer screening.” (Introduction, p4)

<u>Reviewer 3’s Comments</u>:

Comment 1: The first strength/limitation doesn’t really make sense without reading the whole paper and knowing the background challenges- what are the hypothetical scenarios? This is the first strength so should be strong and demonstrate the meaningful contribution this study is contributing to the literature.

Response 1: We thank the reviewer for this comment and we have changed the order of the bullet points because of this. In previous studies on this topic participants who were eligible to attend cancer screening were recruited and asked hypothetically how they might feel if physical activity was discussed. In this study the participants had recently attended screening, which is an improvement on this. We have changed the wording of the bullet point in question to avoid this ambiguity.

- *“This study used data prompted interviews to generate rich qualitative data on cancer screening attendees’ receptivity to physical activity advice.*

- *Participants were recent cancer screening attendees, reducing recall bias and enhancing ecological validity.”* (Strengths and limitations of this study, p3)

Comment 2: I don’t really understand the 4th strength/limitation of the study and it doesn’t really feel like a strength or limitation of your study. Its not really a finding that you made nor is it a major focus for your discussion (which is the appropriate place for this point to be). The limitation could be that the study did not consider whether there were gender and screening type differences in receptiveness to discussing PA.

Response 2: We thank the reviewer for this comment and the suggested alternative. We have edited the bullet point as per the suggestion.

- “*Participants views were considered collectively, and potential differences by gender or screening programme (breast, cervical, bowel) were not explored*.*” (*Strengths and limitations of this study, p3)

Comment 3: Pg 4: “Cancer screening programmes in England bring over 5 million people into contact with a healthcare professional each year, this figure was higher before bowel cancer screening moved to faecal occult blood testing.” Some punctuation is missing here

Response 3: We thank the reviewer for this comment. We have split the sentence into two which hopefully reads better.

“*Cancer screening programmes in England bring over 5 million people into contact with a healthcare professional every year.[8] This figure was higher before bowel cancer screening transitioned to faecal occult blood testing*.” (Introduction, p4)

Comment 4: I actually don’t feel like this aim aligns well with your results- so either you are not achieving your aim with the results that you are presenting or your aim isn’t right for the data you have. From what you’ve presented as results, it feels like the aim of this study is to explore differences in receptivity to advice about physical activity at the time of cancer screening. The second part of the aim statement as it currently stands makes the study feel biased towards finding out why it wont work and is not really addressed as all that is presented in your results. You even mention in the discussion that its not known if doing this would change people’s engagement with screening.

Response 4: We thank the reviewer for this comment, and have amended our aim in line with this comment and that of Reviewer 1.

“*Therefore, the aims of this study were to gain an in-depth understanding of why individual differences in receptivity to physical activity advice exist at cancer screening, and how personal experiences of screening may influence preferences for receiving this advice*.” (Introduction, p4)

Comment 5: How many participants had indicated in the original survey that they were not open to discussing PA? I think this data should be included in table 1.

Response 5: In ‘Conversation Time’ the participants were asked to rate their openness to discussing physical activity on a scale from 1 to 10, 5 times a day for 11 days. We have clarified this in the methods section. Because openness was assessed repeatedly, and varied over time, it was not summarised into a single score or categorised per participant. As such, it is not possible to meaningfully report how many participants were “not open” without considerable discussion and additional quantitative

analysis, which is beyond the scope of this qualitative paper that focused on exploring the variations via data-prompted interviews.

"*A key question within this survey, at each time point, asked them to report their openness, on a rating scale from 0-10, to having a conversation with a healthcare professional about physical activity and cancer prevention. This approach captured fluctuations in receptivity across different moments and contexts, rather than a single static measure. As openness varied within individuals over time, it was not summarised into a single score or categorised per participant*." (Recruitment and Participants, p6)

Comment 6: I know another reviewer raised that all of the quotes are from females and that you felt you had used the best quotes for each section regardless of gender. 12 quotes are used through this results section but they are only from 8 people, with doesn't feel like its potentially truly representative of the sample. Particularly in the communication section, you have 4 quotes but they are only from 2 people. Also none of the quotes are from people that are undergoing screening for bowel cancer. It would be better to have quotes from more people as well as all cancer screening types to make sure the results are representative of the whole sample.

Response 6: We thank the reviewer for this comment. We have revised this section to include quotes from a greater number of participants (from 8 to 12). We have also included more quotes from breast and bowel screening attendees.

"I think, just the process of going through this (cancer screening) pricks your ears up a bit… Because I was going to have this done, it was making me think about it (cancer)" (Bowel screening, Female, #16) (Relevance and convenience, p8)

"On the actual day it is taboo, as far as I'm concerned, but once you get your results..you think…what have I been doing that helps me to avoid it.. you could build in.. a letter or a follow-up phone call. And just say, 'well, you've had all your results..; would you be receptive to having a discussion.'" (Breast screening, Female, #15) (Anxiety during screening, p10)

"*I think it would depend on the person that was doing it. Because I've had three people that have struck me as quite different styles and different people." (Breast screening, Female, #3)* (Communication styles of healthcare professionals, p10)

"I'm like, "Yes, yes. I know this…Yes, exercise good. Yes, I am aware of this"…So I think if it's like tailored to the individual then that's good" (Cervical screening, Female, #4) (Communication styles of healthcare professionals, p11)

Comment 7: Pg 14: "Screening attendees' receptivity could be influenced by the timing of a discussion and by their levels of anxiety throughout screening" This is the first sentence of the conclusion and I think it should be clarified to include PA. E.g. …receptivity to discussing PA….

Response 7: We thank the reviewer for this comment and have amended the sentence as suggested.

"*Cancer screening attendees' receptivity to discussing physical activity could be influenced by the timing of a discussion and by their levels of anxiety throughout screening*." (Conclusion, p16)

VERSION 3 - REVIEW

Reviewer 3

Name Dylke, Elizabeth S

Affiliation The University of Sydney

Date 02-Nov-2025

COI

Thank you for your thoughtful changes in response to my and the other reviewer's previous feedback. I think the changes that you've made have strengthened and clarified the manuscript

Just one minor suggestion:

Pg 4, line 52"…why individual differences in receptivity to physical activity advice exist at cancer screening…"

- This aim statement is an improvement but I'm not sure if you are really looking at "why" individual differences exist. I wonder if the first aim is more "…to gain an in-depth understanding of individuals' receptivity to physical activity advice at cancer screening…"
